# Efficacy of etonogestrel subcutaneous implants versus the levonorgestrel-releasing intrauterine system in the conservative treatment of adenomyosis

**DOI:** 10.1515/med-2024-0914

**Published:** 2024-03-21

**Authors:** Anwen Wei, Xuedong Tang, Wenjuan Yang, Jianqing Zhou, Weili Zhu, Shan Pan

**Affiliations:** Department of Gynecology, Jiaxing Women and Children’s Hospital Wenzhou Medical University, Jiaxing 314051, China

**Keywords:** adenomyosis, etonogestrel, implant, levonorgestrel, dysmenorrhea, menorrhagia

## Abstract

To evaluate the clinical efficacy of etonogestrel subcutaneous implant (ENG-SCI) with that of the levonorgestrel-releasing intrauterine system (LNG-IUD) for adenomyosis treatment. A prospective randomized cohort study was conducted including 108 patients (50 patients in ENG-SCI group and 58 in the LNG-IUD group) with adenomyosis from January 2019 to July 2021. After 3 months of treatment, both ENG-SCI group and LNG-IUD group showed significant improvement in patients’ visual analog scale, pictorial blood loss assessment chart (PBAC), and uterine volume (*P* ＜ 0.05). The uterine volume of patients in LNG-IUD group decreased more significantly than that in the ENG-SCI group since 3 months of treatment. The PBAC score in the LNG-IUD group improved better than that in the ENG-SCI group since 6 months of treatment (*P* ＜ 0.05). No significant difference in the occurrence rate of ideal vaginal bleeding patterns and the hemoglobin levels between the two groups was observed. The ENG-SCI group had a higher probability of weight gain and progesterone-related side effects (*P* ＜ 0.05). Both ENG-SCI and LNG-IUD were effective in treatment of adenomyosis. However, LNG-IUD had a more significant effect in treating adenomyosis-related dysmenorrhea, excessive menstrual flow, anemia, and uterine enlargement, with relatively fewer side effects.

## 1 Introduction

Adenomyosis is a common disease that affects women of childbearing age and has an incidence of 20.9–34.0% on ultrasound [1]. It is primarily characterized by progressive dysmenorrhea, increased menstruation, and enlarged uterus, which negatively affect the quality of life of patients [2]. The treatment chiefly aims to alleviate dysmenorrhea, reduce menstrual volume, and improve the overall quality of life [3].

Historically, hysterectomy has been the traditional surgical treatment for adenomyosis and was considered the standard approach. However, currently there is no ideal conservative treatment for patients who want to retain fertility and maintain physiological function [4]. The levonorgestrel-releasing intrauterine system (LNG-IUD) contains progesterone, which has been widely used in clinical treatment of adenomyosis due to its significant effect [5–7]. The device is a T-shaped device with a tail, which is inserted into the uterus and gradually releases 52 mg of levonorgestrel into the uterine cavity [8]. Progesterone acts locally on the endometrium, blocking estrogen receptors in endometrial glands and stromal cells, thereby inducing endometrial atrophy and degeneration. LNG-IUD can release 20 μg of levonorgestrel daily, and can effectively treat adenomyosis for up to 5 years, so it is a safe and long-lasting treatment option [4,9]. However, issues such as downward displacement and falling off of IUD caused by too large uterine cavity limits its effectiveness in symptom improvement [10,11].

An alternative treatment option is the etonogestrel subcutaneous implant (ENG-SCI), which contains a long-acting progesterone contraceptive. Some studies have demonstrated its efficacy in alleviating dysmenorrhea and menorrhagia [12–16]. The ENG-SCI employs a third-generation progesterone primarily used for contraception. Similar to the LNG-IUD, it contains 68 mg etonogestrel, which suppresses the release of luteotropic hormones and prevents ovulation [12]. The ENG-SCI can relieve adenomyosis symptoms by maintaining higher concentrations of estradiol (E2) than that during the early follicular stage [12]. However, further investigation is warranted to determine whether ENG-SCI is a more efficient option than other conservative approaches for adenomyosis-related dysmenorrhea and menorrhagia. Therefore, the present study was aimed to evaluate and compare the clinical efficacy of ENG-SCI with that of LNG-IUD in improving adenomyosis-associated dysmenorrhea and menorrhagia.

## 2 Materials and methods

### 2.1 Patients

This prospective cohort study included patients with adenomyosis who were treated at our teaching hospital between January 2019 and July 2021.

The inclusion criteria for the study were as follows: (1) patients with adenomyosis symptoms, including varying degrees of dysmenorrhea (secondary) and menorrhagia, and were also diagnosed via 2D-transvaginal color Doppler ultrasound using Sonoline G60S ultrasound imaging system (Siemens, Germany). The following ultrasonic diagnostic criteria were used to diagnose adenomyosis, including an enlarged and asymmetrical uterus, thickened anterior and posterior walls, increased dotted echoes between the muscle walls, ill-defined myometrium heterogeneity, and proliferation of binding bands [17]. (2) Age between 20 and 45 years and without family planning within 1 year.

The exclusion criteria included the following [18]: (1) contraindications for the subcutaneous implantation of ENG-SCI or the placement of LNG-IUD, (2) presence of other endocrine, immune, or metabolic diseases, (3) presence of malignant tumors or uterine precancerous lesions, endometrial hyperplasia, (4) abnormal liver, kidney, and coagulation function or other blood diseases, (5) difficult in follow-up, loss of the intrauterine system (IUD), or early removal of the treatment system, (6) with history of hormonal drug usage within 6 months before treatment, and (7) with fibroid more than 2 cm or submucous fibroids distorting the uterine cavity. Based on the above criteria, 108 patients were included in the study, with 50 in the ENG-SCI group and 58 in the LNG-IUD group.


**Ethical approval:** This study followed the ethical principles outlined in the Helsinki Declaration II and received approval from the Institutional Review Boards of Jiaxing Women’s and Children’s Hospital affiliated to Wenzhou Medical University (approval number: 2018-74). Written informed consent was obtained from each individual participant or their guardian.

### 2.2 Clinical intervention

Eligible patients who have informed consent will be given a generated random number using a computer, with odd numbers assigned to ENG-SCI group and even numbers assigned to LNG-IUD group.

The patients in the ENG-SCI group were treated with etonogestrel implants, produced by the Dutch Ocanon Company. The implant contained 68 mg etonogestrel. The implantation procedure was performed within the first to fifth day of the menstrual period by trained clinical staff. In the LNG-IUD group, the patients received LNG-IUD, manufactured by Bayer Schering Healthcare AG of Germany. The device contained 52 mg levonorgestrel and were inserted according to the manufacturer’s instructions within the fifth to seventh day of the menstrual period. All the procedures were performed by an experienced medical team. The degree of dysmenorrhea was assessed by visual analog scale (VAS), the amount of menstrual blood was evaluated by menstrual diary, and the level of hemoglobin was detected through blood routine. We requested the participants to visit our clinic every 3 months for at least 1 year, and special designed table were used to record everyone’s information.

### 2.3 Study outcomes

The improvement in dysmenorrhea was evaluated using the VAS score, which ranges from 0 to 10. Higher scores indicate severer pain. The patients were advised to mark the line mainly based on their maximum perception of pain during the last menstrual cycle [19].

Vaginal bleeding patterns were categorized as follows according to World Health Organization-recommended definition [20]: amenorrhea (no bleeding or spotting within 90 days), sparse bleeding (<3 episodes of bleeding or spotting within 90 days), normal frequency (3–5 episodes of bleeding or spotting within 90 days), frequent bleeding (>5 episodes of bleeding or spotting within 90 days), and prolonged bleeding (continuous bleeding or spotting lasting more than 14 days). The ideal vaginal bleeding pattern included <5 episodes of bleeding or dripping within 90 days [13,21].

The menstrual volume changes were assessed by menstrual diary using a pictorial blood loss assessment chart (PBAC) developed by Higham and his friends [22]. Patients were instructed to fill in the menstrual diary at the first time, and were suggested to use the same brand of sanitary pads and replace them when they get the same dirty to minimize subjectivity [23]. The uterine volume was measured by Doppler transvaginal ultrasound, and was calculated by length × anteroposterior diameter × transverse diameter × 0.52 [19]. Hemoglobin levels were measured via venous blood sampling to serve as an auxiliary standard for evaluating the degree of menorrhagia. In addition, the adverse events after treatment were recorded throughout the study, such as weight gain (increase ≥5 kg) [10], acne, new ovarian cysts on transvaginal ultrasound (TVS), contraceptive failure, and progesterone-related side effects (including breast and body pain, headache, dizziness, mood changes, and hair loss). Acne was determined through consultation with a dermatologist, new ovarian cysts were determined by an ultrasound doctor based on vaginal ultrasound and comparison with pre-treatment vaginal ultrasound results. Adverse reactions related to progesterone were determined based on the patient’s complaint compared to pre-treatment. All the participants were followed at the 3rd, 6th, 9th, and 12th months after the treatment.

### 2.4 Sample size

The sample size in our study was estimated using the following method [18]: the sample size was calculated mainly based on the improvement of VAS before and after treatment. Previous studies reported that LNG-IUD improved dysmenorrhea with the decrease of VAS from 81.2 to 55.2%, and ENG-SCI from 76.2 to 15.5% after treatment for 3 months [10,14]. Using a two-sided chi-square (*χ* ^2^) test (*α* = 0.05), we calculated the sample size for at least 90 patients in two groups (45 in each group). Additionally, we assumed that the missed follow-up rate was 15%, so finally at least 104 patients (52 in each group) must be included.

### 2.5 Statistical analysis

The data were analyzed using IBM SPSS Statistics (Version 22.0, IBM Corp, Armonk, NY). Continuous variables with a normal distribution were presented as mean ± standard deviation, whereas those without a normal distribution were described as median with interquartile range. To compare two groups with normally distributed quantitative data, independent *t*-test was performed. For non-normally distributed quantitative data, Mann–Whitney *U*-test was employed. Chi-squared test was used to compare categorical data. Kruskal–Wallis test with the *H*-test for multiple comparisons was applied when comparing three or more groups with non-normally distributed data. Conversely, for normally distributed data, one-way analysis of variance with the least significant difference test was used for multiple comparisons. A *P*-value of <0.05 was considered statistically significant.

## 3 Results

### 3.1 Clinical baseline characteristics of patients in two groups before treatment

In total, 108 patients consented to participate in the study, with 50 patients receiving the ENG-SCI treatment and 58 receiving the LNG-IUD treatment. The results showed that no significant differences were observed between the two groups in terms of age, education years, gravida, parity, abortion, VAS score, hemoglobin, uterine volume, and PBAC score (Table 1).

**Table 1:** Baseline characteristics in two groups

Group	Age	Gravida	Parity	Abortion	Education (years)	Uterine volume (cm^3^)	VAS	Hemoglobin (g/L)	PBAC
LNG-IUD (*N* = 58)	38.8 ± 3.4	4.0 (3.0–4.0)	1.0 (1.0–2.0)	3.0 (2.0–3.0)	15.0 (13.0–16.0)	121.5 ± 15.3	7.0 (5.0–7.0)	97.8 ± 11.6	120.2 ± 15.6
ENG-SCI (*N* = 50)	38.3 ± 4.6	3.0 (3.0–4.0)	1.0 (1.0–2.0)	2.0 (1.0–3.0)	14.0 (13.0–15.0)	120.9 ± 16.4	7.0 (6.0–7.0)	100.2 ± 12.3	117.2 ± 14.5
*P*	0.525	0.186	0.396	0.105	0.262	0.847	0.475	0.306	0.311

VAS, visual analog scale; LNG-IUD, levonorgestrel intrauterine system; ENG-SCI, etonogestrel subcutaneous implant; PBAC, pictorial blood loss assessment chart.

### 3.2 Comparison of clinical efficacy before and after the treatment in ENG-SCI and LNG-IUD group

The ENG-SCI can prominently improve the clinical symptoms of patients with adenomyosis, such as dysmenorrhea and menorrhagia. After 3 months of treatment, the patient’s VAS score, PBAC score, and uterine volume all showed a significant decrease (*P* < 0.05) (Table 2). The VAS score decreased from 7.0 (5.0–7.0) to 4.0 (3.0–4.5), the PBAC score decreased from 117.2 ± 14.5 to 70.2 ± 12.9, and the uterine volume decreased from 120.9 ± 16.4 to 102.3 ± 13.4 cm^3^, and showed a continuous downward trend during the subsequent follow-up. After 6 and 12 months of treatment, the patient’s hemoglobin levels increased from 100.2 ± 12.3 to 108.5 ± 8.4 and 125.4 ± 9.5 g/L, respectively. Similar results were observed in LNG-IUD group, as shown in Table 3.

**Table 2:** Outcomes after subcutaneous implantation of etonogestrel

Outcomes	Baseline	Follow-up time after implantation (months)				*P*
		3	6	9	12	
VAS	7.0 (6.0–7.0)	4.0 (3.0–4.5)^a^	3.0 (2.0–3.5)^a^	2.0 (2.0–3.0)^ab^	2.0 (1.0–2.5)^abc^	＜0.01
PBAC	117.2 ± 14.5	70.2 ± 12.9^a^	52.6 ± 9.6^ab^	40.3 ± 7.8^abc^	36.6 ± 5.3^abcd^	＜0.01
Hemoglobin (g/L)	100.2 ± 12.3	NA	108.5 ± 8.4^a^	NA	125.4 ± 9.5^ac^	＜0.01
Uterine volume (cm^3^)	120.9 ± 16.4	102.3 ± 13.4^a^	96.3 ± 8.9^ab^	86.7 ± 7.6^abc^	77.3 ± 7.9^abcd^	＜0.01

^a^Compared to baseline (*P* < 0.05); ^b^Compared to 3 months after implantation (*P* < 0.05); ^c^Compared to 6 months after implantation (*P* < 0.05); ^d^Compared to 9 months after implantation (*P* < 0.05); VAS, visual analog scale; NA, not applicable; PBAC, pictorial blood loss assessment chart.

**Table 3:** Outcomes after placement of levonorgestrel intrauterine system

Outcomes	Baseline	Follow-up time after placement (months)				*P*
		3	6	9	12	
VAS	7.0 (5.0–7.0)	4.0 (4.0–5.0)^a^	3.0 (2.0–3.0)^ab^	2.0 (2.0–3.0)^ab^	2.0 (1.0–2.0)^abc^	＜0.01
PBAC	120.2 ± 15.6	72.3 ± 13.6^a^	48.5 ± 8.8^ab^	36.4 ± 8.1^abc^	24.2 ± 6.0^abcd^	＜0.01
Hemoglobin (g/L)	97.8 ± 11.6	NA	110.7 ± 11.3^a^	NA	127.3 ± 10.7^ac^	＜0.01
Uterine volume (cm^3^)	121.5 ± 15.3	90.3 ± 12.9^a^	75.6 ± 8.8^ab^	71.53 ± 7.7^abc^	65.6 ± 6.8^abcd^	＜0.01

^a^Compared to baseline (*P* < 0.01); ^b^Compared to 3 months after placement (*P* < 0.01); ^c^Compared to 6 months after placement (*P* < 0.01); ^d^Compared to 9 months after placement (*P* < 0.01); VAS, visual analog scale; NA, not applicable; PBAC, pictorial blood loss assessment chart; LNG-IUD, levonorgestrel intrauterine system.

### 3.3 Comparison of clinical efficacy between ENG-SCI and LNG-IUD group

**Figure 1:**
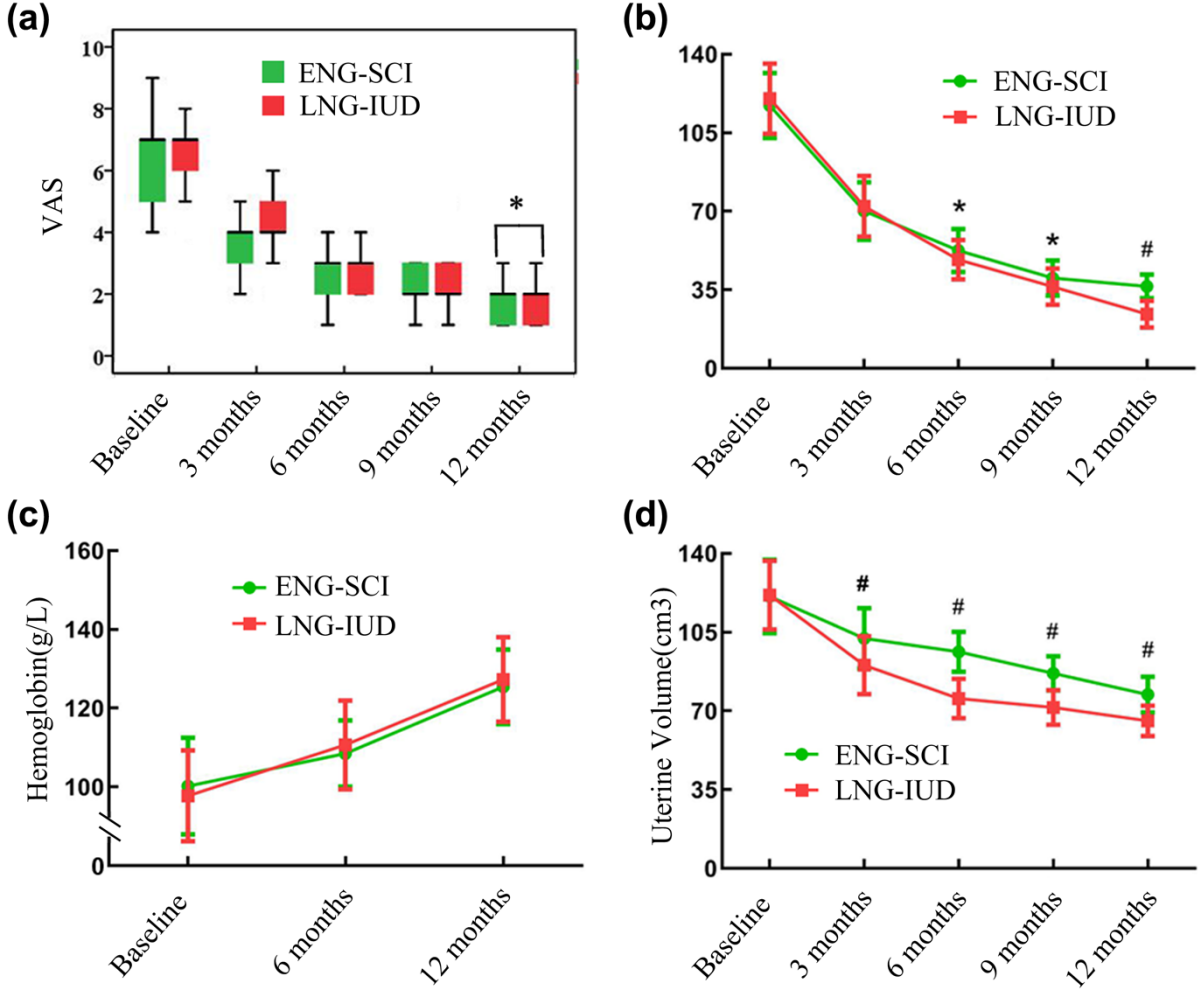
Comparison of the treatment efficacy in ENG-SCI and LNG-IUD group. *, *P* < 0.05; #, *P* < 0.001. ENG-SCI: etonogestrel subcutaneous implant; LNG-IUD: levonorgestrel-releasing intrauterine system; VAS, visual analog scale; PBAC, pictorial blood loss assessment chart. The therapeutic effects between two groups, regarding VAS score (a), PBAC (b), hemoglobin (c), and uterine volume (d).

The research results showed that there was no significant difference in VAS scores between the two groups in the first 9 months, and until 12 months after treatment, the VAS score of the LNG-IUD group was significantly lower than ENG-SCI group (Figure 1a, *P* < 0.05). There was no significant difference in PBAC scores between the two groups after 3 months of treatment; however, the PBAC scores of the LNG-IUD group were significantly lower than ENG-SCI group from 6 to 12 months after treatment (Figure 1b, *P* < 0.05). There was no significant difference in hemoglobin levels between the two groups after treatment (Figure 1c). The uterine volume in the LNG-IUD group was significantly lower than ENG-SCI group from 3 to 12 months after treatment (Figure 1d). The rate of ideal vaginal bleeding patterns increased gradually over 1 year treatment in both groups. However, no statistically significant differences were observed between the two groups (Table 4).

**Table 4:** Rate of ideal vaginal bleeding patterns in two groups after treatment

Group	Follow-up time after placement (months)			
	3	6	9	12
ENG-SCI (*N* = 50)	25 (50.0%）	36 (72.0%)	39 (78.0%)	43 (86.0%)
LNG-IUD (*N* = 58)	25 (43.1%)	42 (72.4%)	44 (75.9%)	55 (94.8%)
*P*	0.476	0.962	0.794	0.116

LNG-IUD, levonorgestrel intrauterine system; ENG-SCI, etonogestrel subcutaneous implant.

### 3.4 Adverse events

The adverse events that occurred during the follow-up period included weight gain, acne, ovarian cysts, and progesterone-related side effects such as breast and whole-body pain, headache, dizziness, hair loss, and mood changes (Table 5). Weight gain or loss of ≥5 kg was considered a significant weight change. During the 12 months of treatment, the ENG-SCI group exhibited significantly higher incidence of body weight gain than the LNG-IUD group (32.0 vs 9.1%, *P* = 0.002). The ENG-SCI group also showed significantly higher incidence of progesterone-related side effects (38.0%) than the LNG-IUD group (14.5%, *P* = 0.004). However, there were no significant differences in the incidence of acne and ovarian cysts between the two groups.

**Table 5:** Adverse events after treatment in two groups

	ENG-SCI (*N* = 50)	LNG-IUD (*N* = 58)	*P*
Weight gain (≥5 kg)	16 (32.0%)	5 (9.1%)	0.002
Acne	10 (18.0%)	5 (8.6%)	0.090
New ovarian cyst	8 (16.0%)	8 (14.5%)	0.749
Contraceptive failure	0 (0)	0 (0)	—
Progesterone-related side effects^*^	19 (38.0%)	8 (14.5%)	0.004

LNG-IUD, levonorgestrel intrauterine system; ENG-SCI, etonogestrel subcutaneous implant.

*Symptoms include breast and body pain, headache, dizziness, mood changes, hair loss, etc.

## 4 Discussion

Adenomyosis is a chronic estrogen-dependent disease which is characterized by the existence of endometrial glands and stroma within the myometrium. The main manifestations of adenomyosis are increased uterine bleeding, progressive dysmenorrhea, chronic pelvic pain, and spherically enlarged uterus size [24,25]. The surgical treatments include hysterectomy, excision of uterine adenomyosis lesions, and endometrial ablation. Traditionally, hysterectomy was considered as the main radical treatment [26]. Other non-invasive treatments are increasingly offered, including gonadotropin-releasing hormone analogs, levonorgestrel-releasing intrauterine devices, danazol, high-intensity focused ultrasound, and other oral contraceptives [23]. More recently, a few observational studies used the etonogestrel subdermal implant, a long-term contraceptive device, for the treatment of adenomyosis [16]. However, to our knowledge, this is the first study that compare the efficacy of the etonogestrel subdermal implant with LNG-IUD.

Our present study showed that both LNG-IUD and etonogestrel subdermal implant were effective in cure of adenomyosis in terms of reducing uterine bleeding, improving hemoglobin, and alleviating dysmenorrhea, but LNG-IUD group showed superiority to etonogestrel subdermal implant regarding reduced uterine volume size, and similar results were also observed in Shaaban’s study which compared the LNG-IUD to low-dose combined oral contraceptive for the treatment of adenomyosis [18].

Progestin is believed to have a direct effect on the adenomyosis foci and make them hypotrophy subsequently [24]. Both LNG-IUD and ENG-SCI release progestin, but differently, the former was attributed to its direct action on down-regulation of estrogen receptor of uterine endometrial and its decidualization to reduce menstrual flow [27], and its pain-relieving ability was obtained by reducing prostaglandin production in the endometrium and inducing atrophy of adenomyosis foci [19], while the latter was based on ovulation inhibition to eliminate the estrogen-ovulation peak, decreasing the average level of estrogen in the menstrual cycle, thus thinning the endometrium. Previous study have also demonstrated that the ENG-SCI may relieve the pain by inhibition ovulation leading to reduced levels of endogenous thromboxane A2, vasopressin, oxytocin levels, and prostaglandin I2 [13].

The relief of dysmenorrhea was the most important outcome in the present study. Our study showed that the VAS score in both LNG-IUD group and ENG-SCI group was significantly reduced after 3 months of treatment, these were consistent with previous studies [10,13,14]. In Wu and Nie’s studies [13,14], they observed declines of VAS score from 7.62 ± 0.74 to 1.55 ± 0.42, 1.45 ± 0.35 and from 10 (10,10) to 0 (0,1), 0 (0,1) at time points of 3 and 12 months after ENG-SCI implantation, respectively. In our study, the VAS score in ENG-SCI were 7.0(5.0–7.0), 4.0(3.0–4.5), and 2.0(1.0–2.5) at 3 and 12 months after treatment; we did not observe such great reduction, perhaps because of the disease heterogeneity and population diversity, and also because these two studies were of small sample size. Li’s study showed that LNG-IUD placement could reduce VAS score from 8.1 ± 0.9 to 5.5 ± 2.4, 3.2 ± 2.1 after 3 and 12 months of insertion [10], our study also demonstrated similar results. When we compared the VAS scores between LNG-IUD group and ENG-SCI group, there was no statistical significance until 12 months of treatment, indicating that LNG-IUD group showed much lower VAS score than ENG-SCI group.

Menorrhagia was another important clinical manifestation of adenomyosis which can result in anemia, and it is a critical reason of hysterectomy. So we use the changes in hemoglobin levels as an indicator of treatment effect. As was shown in previous study, the menstrual flow decreases significantly from 100.00 to 56.32 ± 36.21% and 41.12 ± 32.45% at 3 and 12 months after implantation of ENG-SCI [16], and the hemoglobin level prominently improved from 3 months of ENG-SCI implantation [13,14]. Our results were in accord with them. In addition, Shaaban et al. found that the number of sanitary pads used per day during menstrual period decreased from 6.29 ± 0.69 to 2 ± 1.44 after 6 months insertion of LNG-IUD [18]. The hemoglobin level was reported to increase from 93.2 ± 13.9 to 100.9 ± 21.9 [10]. All these results were in agreement with our present study; in addition, our study also showed that LNG-IUD is more efficient in decrease in menstrual flow compared to ENG-SCI.

The adverse events were the main concerns of patients, which mainly included weight gain, acne, ovarian cyst, and progestin-related side effect, such as body swelling, headache, dizziness, emotional changes, and hair loss [28]. In our study, weigh gain and acne were the most common, with incidence of 32.0 and 18.0% in ENG-SCI group, respectively, which were significantly higher than LNG-IUD group (9.1 and 8.6%), the reason may be attributed to the different action-pathways of the two devices. In LNG-IUD group of our study, the incidence of weight gain was almost consistent with Li’s report [10], but there was another study, which demonstrated that weight gain may not be attributable to LNG-IUD, because weight gain was also observed in non-hormonal IUD users [29]. The acne incidence was much higher than previous study, which may be due to the difference of body constitution. In Blumenthal’s study, they observed ENG-SCI-related adverse events of weight gain (11.8%) and acne (11.4%). Besides, Chunfen et al. reported that the weight gain rate was 28.7% (31/150) [30], which was in line with our present results. The incidence of ovarian cyst was 16.0 and 14.5% in ENG-SCI and LNG-IUD group, respectively, and of no statistical significance. Previous study showed that LNG-IUD had little effect on ovarian function and did not compromise oocyte quality and follicular development, and the occurrence of amenorrhea was mainly due to the local effect of LNG-IUD [31,32]. However, Tasci found a significant increase of follicle-stimulating hormone (FSH) in 69% LNG-IUD users at 1 year of insertion, but the volume of ovaries was not changed [32]. In 2018, Wu’s study suggested that the levels of FSH, luteinizing hormone (LH), and E2 after 3, 6, and 12 months of implantation were similar to baseline, indicating that etonogestrel had no prominent effects on the female hormone levels [14]. We regret that we were unable to evaluate the changes in patient sex hormones (FSH and LH) during the treatment process in our study, and more studies are needed to confirm this.

The strength of our study was that we were the first to compare the therapeutic effectiveness of ENG-SCI and LNG-IUD device on adenomyosis. There were also some limitations, including a short follow-up period and lacking of monitoring of CA125 and female endocrine hormones during treatment. A larger prospective multicenter study is warranted to confirm our findings.
